# DNA Metabarcoding Focusing on Eukaryote Communities on Langhovde Glacier, East Antarctica

**DOI:** 10.1111/1758-2229.70117

**Published:** 2025-07-02

**Authors:** Hiroto Kajita, Ken Kondo, Shin Sugiyama, Yasuhide Nakamura, Sakae Kudoh, Koji Umeda

**Affiliations:** ^1^ Graduate School of Science and Technology, Hirosaki University Hirosaki, Aomori Japan; ^2^ National Institute of Polar Research, Research Organization of Information and Systems Tachikawa, Tokyo Japan; ^3^ Institute of Low Temperature Science, Hokkaido University Sapporo Hokkaido Japan; ^4^ Graduate School of Environmental Science, Hokkaido University Sapporo Hokkaido Japan; ^5^ Graduate School of Environmental Studies Nagoya University Nagoya Aichi Japan; ^6^ Arctic Research Center Hokkaido University Sapporo Hokkaido Japan; ^7^ Estuary Research Center Shimane University Matsue Shimane Japan; ^8^ Department of Botany National Museum of Nature and Science Tsukuba Ibaraki Japan; ^9^ Polar Science Program The Graduate Institute for Advanced Studies, SOKENDAI Tachikawa Tokyo Japan

**Keywords:** 18S rRNA, antarctic glacier, ice algae, snow algae, supraglacial water

## Abstract

Ecological structures and habitats of eukaryote communities in supraglacial environments have attracted attention because of their unique biodiversity and potential impact on glacial surface melt. In this study, we investigated the microbial community on Langhovde Glacier in East Antarctica, wherein few molecular biological studies have been conducted. We performed a comprehensive environmental DNA analysis and dissolved ion measurements focusing on various types of supraglacial waters scattered on Langhovde Glacier, as well as ponds in the adjacent off‐ice area. 18S rRNA gene analysis revealed the presence of diverse eukaryotic taxa on the glacier, including Chlorophyta, Chrysophyceae, Cercozoa, Choanoflagellatea and Streptophyta. Distinct ecological structures were observed between large perennial supraglacial lakes and small transient supraglacial puddles on the glacier. Moreover, microbial diversity was greater in the off‐ice ponds with elevated concentrations of dissolved ions. Only a limited number of eukaryotic gene sequences were shared on‐ and off‐ice sites, and many of the gene sequences detected on Langhovde Glacier matched those from remote snow and ice fields worldwide. These results highlight the cosmopolitan nature of ice/snow algae and suggest that the physicochemical properties of the supraglacial water environment play a crucial role in shaping microbial diversity on glacier surfaces.

## Graphical abstract

Based on comprehensive 18S rRNA gene analysis, this study characterises the eukaryotic communities inhabiting the supraglacial environments of Langhovde Glacier, East Antarctica, in relation to the associated aquatic chemistry. Genetic differences between organisms found in the surrounding ice‐free areas and those in distant snow and ice fields are also discussed.

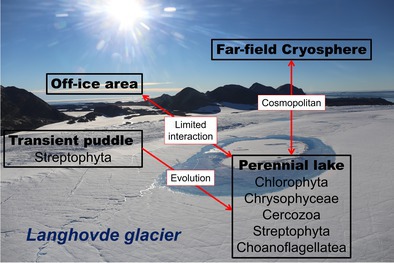

## 1 Introduction

Antarctica is the coldest, windiest and driest continent on Earth, which forms an unfavourable environment for life. Nevertheless, freshwater ecosystems harbouring bacteria, archaea, and some eukaryotes are found along the coast in a highly oligotrophic environment (Beyer and Bölter 2002; Verleyen et al. 2012; Kudoh and Tanabe 2014). Relatively long‐lived ponds are colonised by diverse microorganisms (Chaya et al. 2019; Hirose et al. 2020), some of which develop into microbial mats and biofilms (Imura et al. 2003; Nakai et al. 2012). On the ice sheet, biological diversity in cryoconite holes has been studied and reported as a unique microbial community (Millar et al. 2021). However, the microbiological data from glacier surface layers in Antarctica remain scarce because of the logistical challenges of fieldwork in these environments (Webster‐Brown et al. 2015; Weisleitner et al. 2020). As such, the identity and origin of organisms—particularly those inhabiting the seemingly barren, colourless glacier surfaces in Antarctica—are still poorly understood.

The hydrological environment on glaciers is inherently unstable because of glacial movement and is characterised by extremely low concentrations of dissolved ions, including nutrients. Despite these oligotrophic and harsh conditions, ice/snow algae, primarily belonging to Chlorophyta and Streptophyta, have been reported (Hoham and Remias 2020). Some of them are rare primary producers in glacier surface ecosystems, playing key roles in nitrogen and carbon cycles (Hoham and Remias 2020). Additionally, they influence glacier melt dynamics by reducing albedo, thereby affecting the stability of ice sheets (Onuma et al. 2020; Cook et al. 2020; Hotaling et al. 2021). Therefore, it is essential to deepen our understanding of microbial community structures in relation to the geochemical characteristics of the Antarctic glacial surface.

Previous studies have shown that many of the ice/snow algae found on Antarctic glaciers are cosmopolitan, and their broad geographic distribution and propagation have attracted ecological attention (Segawa et al. 2018, 2023). Although these studies have primarily focused on comparisons among remote ice and snow fields, few have examined microbial communities on nearby nunataks or analysed community variation within individual glaciers. This hinders our understanding of the local‐scale dispersal and establishment process of ice/snow algae. In this study, we report the distribution of eukaryotic microbes in supraglacial meltwaters on Langhovde Glacier, East Antarctica, using DNA metabarcoding analysis. We compare the microbial community structure with those from the surrounding off‐ice area and previously studied ice/snow fields worldwide, aiming to estimate the developmental mechanism of the microorganism community on the glacier surface.

## 2 Study Site and Sampling Protocols

**FIGURE 1:**
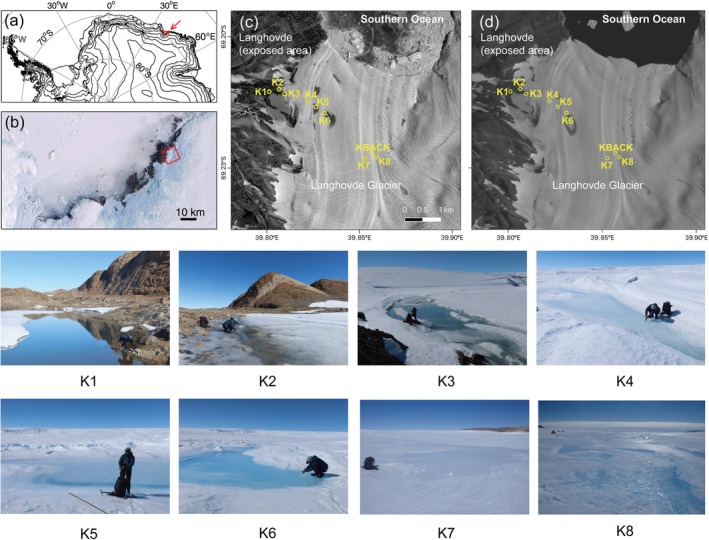
The study area and sampling sites. (a) Antarctica and the location of Lützow–Holm Bay (red box, upper right). (b) Satellite image (Landsat Imagery Mosaic Antarctica) of Lützow–Holm Bay. (c) A Landsat 8 OLI image acquired on 1 February 2021 shows the sampling site on and the surrounding off‐ice area (K1–K3) and supraglacial waters on Langhovde Glacier (K4–K8). K4 and K8 are small transient supraglacial puddles, whereas K5 and K6 are relatively large perennial supraglacial lakes; K7 is a supraglacial stream covered with thin ice. The pictures of sampling sites are also shown in this figure. (d) A Landsat 8 OLI image was acquired on 1 February 2018. The location corresponding to the sampling sites is marked.

Langhovde (69°14′ S, 39°40′ E) is located approximately 30 km south of the Japanese Syowa Station on the Soya Coast, along the eastern margin of Lützow–Holm Bay in East Antarctica. Langhovde Glacier is an outlet glacier that drains the East Antarctic Ice Sheet into Lützow–Holm Bay through a 3‐km wide ice front, with a flow rate of approximately 150 m/year (Figure 1) (Sugiyama et al. 2014). The lower 10 km of the glacier is bound by exposed bedrock on the western side and ice on the eastern side. During summer, the glacier surface becomes exposed as strong katabatic winds inhibit snow deposition. Near its terminus, the glacier transitions into an ice shelf that extends approximately 1–3 km from the calving front. The ice shelf is approximately 230–430 m thick and is in hydrostatic equilibrium (Minowa et al. 2021; Sugiyama et al. 2014). The glacier surface is relatively flat and level in the lower reaches; however, the surface elevation rises by 40 m from the grounding line to a point 500 m inland (Fukuda et al. 2014; Sugiyama et al. 2014).

Surface water samples were collected on 27 January 2022, during the 63rd Japanese Antarctic Research Expedition (2021–2022), from two ponds in the off‐ice area (K1–K2), one site at the western margin of the glacier adjacent to the off‐ice area (K3), and five supraglacial water sites on Langhovde Glacier (K4–K8). On Langhovde Glacier, channels and small lakes generated by melting ice appear every summer (Figure 1). Satellite imagery shows that most of these water bodies freeze over during winter. However, several large lakes reappear in nearly the same location every summer, and their outlines remain visible even during winter (Figure 1) (Langley et al. 2016). Among the sampling sites, K4 and K8 are small transient supraglacial puddles formed from seasonal surface meltwater. K7 is a surface stream originating from the upper reaches of the glacier. K5 and K6 are parts of a perennial supraglacial lake that may not entirely freeze even in winter (Figure 1) (Langley et al. 2016).

Between 0.1 and 1.0 L of surface water was filtered on‐site using a Sterivex filter unit (0.45 μm pore size). The Sterivex filters were frozen (< −20°C) with RNA*later* (ThermoFisher Scientific) and stored for 3 months until DNA analysis. The filtrate was bottled and stored in a cool environment (ca. 10°C) during the fieldwork and in a refrigerator (4°C) for 6 months before subsequent chemical analysis in Japan. pH was measured in situ using a portable pH meter (HORIBA, LAQUAtwin pH‐33B). To evaluate contamination during sampling and analysis, a water‐impermeable Sterivex filter unit was prepared at the KBACK site on Langhovde Glacier (Figure 1) and stored in the same manner as the environmental samples (K1–K8).

## 3 Analytical Procedures

### 3.1 DNA Analysis

DNA was extracted from the Sterivex filters (K1–K8) in a specialised and sterilised laboratory of Bioengineering Lab Co. Ltd. for modern environmental DNA studies using strict contamination prevention protocols. The filtered sample was crushed at 1500 rpm for 2 min using a Shake Master Neo after adding Lysis Solution (NIPPON GENE Co. Ltd.) to the filter paper. DNA was purified from the aliquoted supernatant solution using the MPure 12 System and MPure Bacterial DNA Extraction Kit (MP Bio Co. Ltd.) The library was constructed by two‐step tailed PCR, and the quality was checked using Fragment Analyzer and dsDNA 915 Reagent Kit (Advanced Analytical Technologies Inc.). For deep amplicon sequencing, 18S rRNA analysis was performed using the primer sets 1422F and 1642R, which feature broad coverage of eukaryotic microorganisms (Wang et al. 2014). The nucleotide sequence of these primers, including the Illumina adapter for indexing, was as follows (annealing sequences are underlined).

1422F (5′ → 3′):

ACACTCTTTCCCTACACGACGCTCTTCCGATCT‐ATAACAGGTCTGTGATGCC.

1642R (5′ → 3′):

GTGACTGGAGTTCAGACGTGTGCTCTTCCGATCT‐CGGGCGGTGTGTACAAAGG.

PCR was performed using the following thermal cycle conditions: 2 min of initial denaturation at 95°C, 30 cycles of denaturation for 30 s at 94°C, 30 s of primer annealing at 55°C, 30 s of primer extension at 72°C, with a final extension of 5 min at 72°C. PCR reactions were run in 10 μL volumes using Ex Taq HS DNA polymerase (Takara Bio Co., Japan). PCR blanks were amplified alongside the samples to check for contamination. According to the manufacturer's guidelines, the PCR product was purified using AMPure XP (Beckman Coulter Inc.). Next‐generation DNA sequencing was performed by Bioengineering Lab Co. using the Illumina MiSeq platform with a Reagent Kit v3 to yield two paired reads of each 300 bp long. The fastx_barcode_splitter tool in the FASTX Toolkit (ver. 0.0.14) was used to extract the sequence. The primer sequence, chimeric sequences, and noisy sequences were removed using DADA2, originally an R package wrapped by Qiime 2 (ver 2023.2). Subsequently, representative sequences and ASV tables were obtained as output. Using the feature‐classifier plugin, we compared the obtained representative sequences with the 97% OTU of SILVA (ver. 132) and estimated the phylogeny. The gene sequences were aligned using the ClustalW alignment program implemented in the Mega 11 software (Tamura et al. 2021), with related species published in the GenBank database. Amplicon sequencing variants (ASVs) were assigned to higher taxa (phylum‐ or class‐level taxa), according to Adl et al. (2018) and Nakamura et al. (2019) (Supporting Information). A phylogenetic maximum likelihood tree, including bootstrap probabilities (500 samples) among each phylotype, was constructed using MEGA 11 software (Tamura et al. 2021).

### 3.2 Water Chemical Analysis

Major dissolved ions in the filtered water samples (K1–K8) were analysed using ion chromatography (Eco IC, Metrohm AG). Concentrations of major dissolved cations (Na^+^, K^+^, Ca^2+^, Mg^2+^, NH_4_^+^) and anions (Cl^−^, NO_3_^−^ and SO_4_^2−^) were quantified. The analytical error for the ion concentration measurements was to be within ±0.1%–0.5% (1*σ*) as estimated from the reproductivity of standard solutions. The detection limit for each ion was 0.01 ppm.

### 3.3 Statistical Analysis

Principal component analysis (PCA) was performed on Hellinger‐transformed total read counts of each higher taxonomic group to evaluate pairwise similarities among sampling sites, following the method described by Legendre and Legendre. Additionally, Spearman's rank correlation coefficients were calculated to assess the relationships between the total read abundances of higher taxonomic groups and measured water quality parameters.

## 4 Results

**FIGURE 2:**
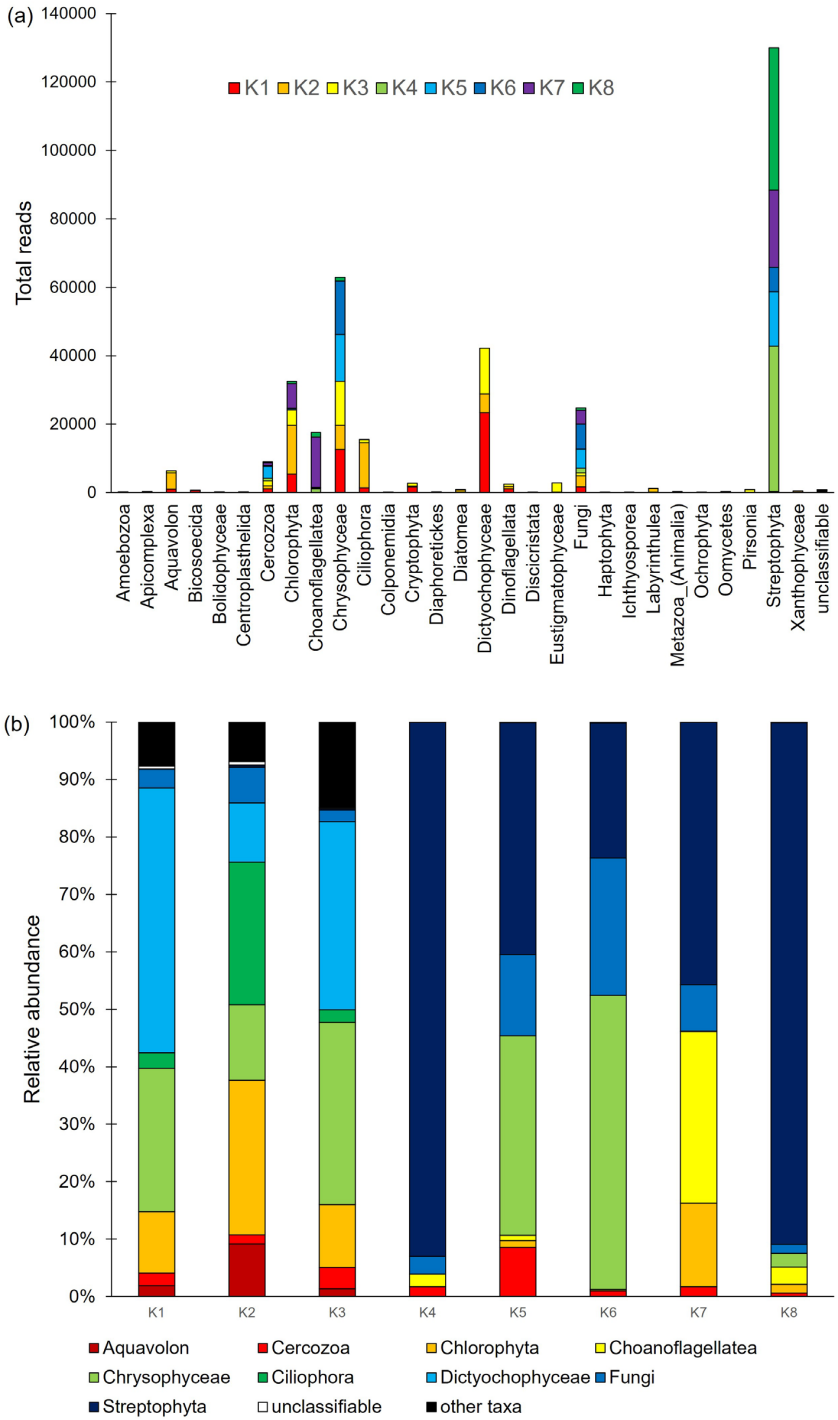
Overall eukaryote community structures of collected samples represented by (a) total read for each higher taxon and (b) relative abundance of each higher taxon per sample site.

**FIGURE 3:**
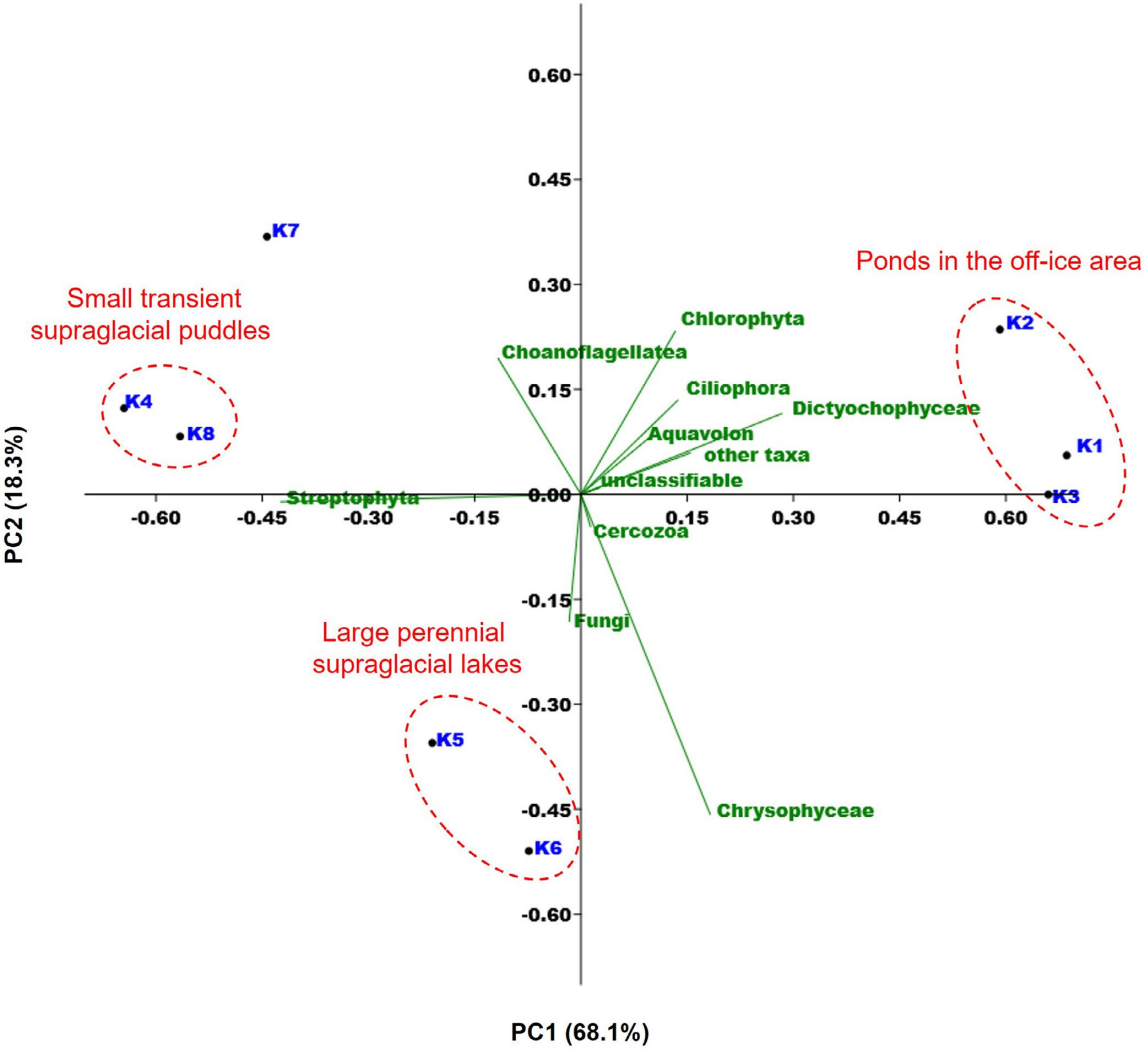
Similarities among the eukaryotic community on and around Langhovde Glacier based on principal component analysis for total reads of the major higher taxonomic groups.

**FIGURE 4:**
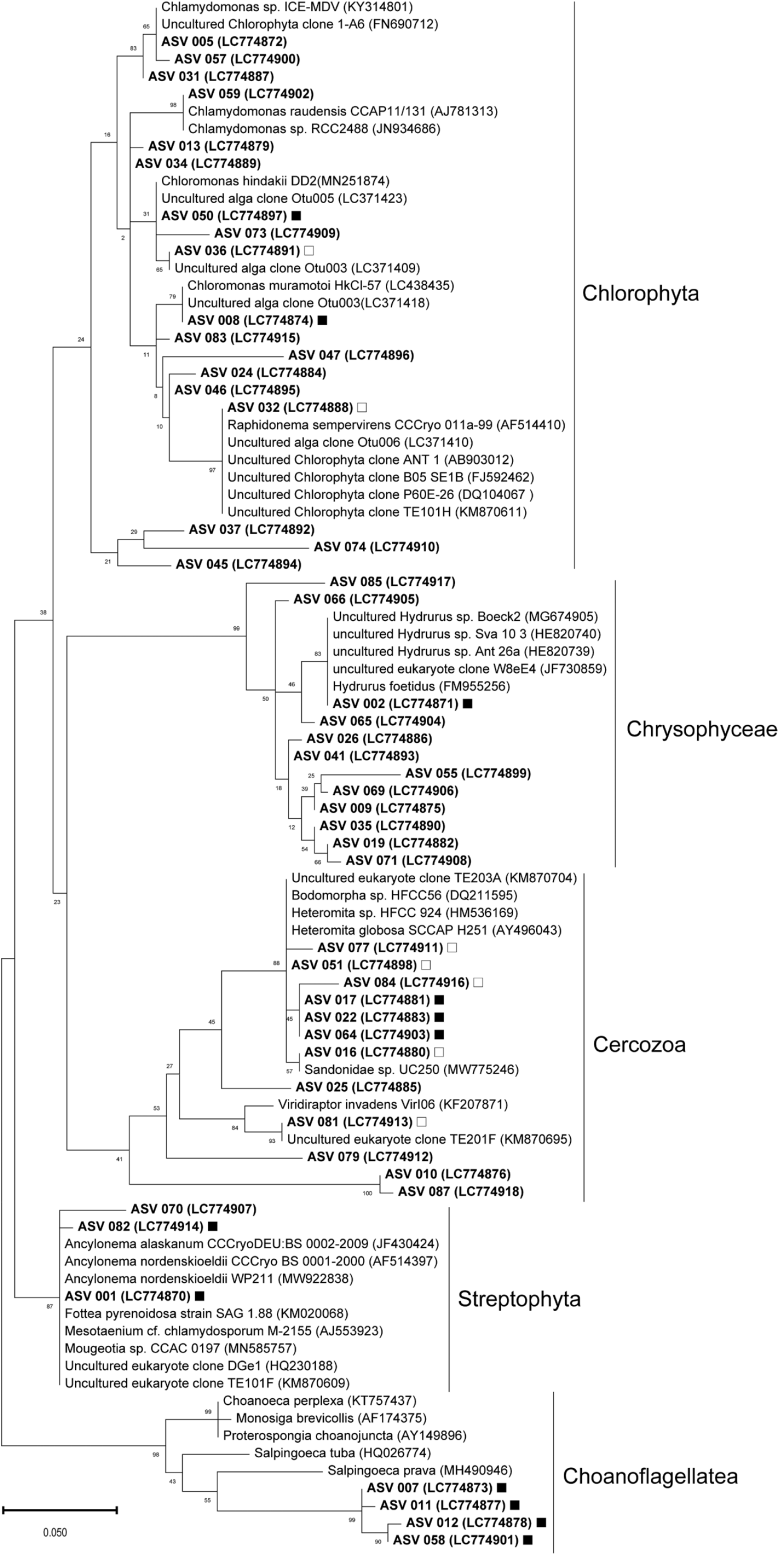
Phylogenetic tree of Chlorophyta, Chrysophyceae, Cercozoa, Streptophyta and Choanoflagellatea clones. Only ASVs with a total lead count of 100 or more were listed. The DNA database accession numbers for described species are in parentheses. The bar indicates evolutionary distance. The bootstrap value of each node is shown. Sequences detected on Langhovde Glacier are marked with a solid square. Sequences detected on Langhovde Glacier and the off‐ice area are marked with an open square. Sequences detected only in the off‐ice area are with no mark.

A total of 346 ASVs were identified across all samples (Supporting Information). Although several ASVs were also detected in the blank control (KBACK), they were not consistently observed in the environmental samples (K1–K8), indicating minimal contamination during sampling. Most ASVs were classified into 29 supergroup‐level topologies (Figure 2a; Supporting Information). Among these, the substantial contributors to relative abundance across the samples (K1–K8) included Aquavolon, Cercozoa, Chlorophyta, Choanoflagellatea, Chrysophyceae, Ciliophora, Dictyochophyceae, Fungi and Streptophyta (Figure 2b). PCA based on these higher taxonomic groups revealed distinct patterns in eukaryotic community composition associated with hydrologic characteristics at each site (Figure 3). In particular, on‐glacier samples (K4–K8) exhibited higher relative abundances of Chlorophyta, Chrysophyceae, Cercozoa, Streptophyta and Choanoflagellatea compared with the off‐ice sites adjacent to the glacier (K1–K3). Notably, although K4 and K8 are approximately 3 km apart, they exhibited highly similar community compositions, both being dominated by Streptophyta, which accounted for more than 90% of the total reads. Sites K5 and K6 were characterised by relatively high abundances of Chrysophyceae, whereas K7 was dominated by Chlorophyta and Choanoflagellatea. These taxonomic assignments are summarised in a phylogenetic tree with presence/absence information on each sampling site and have been deposited in the DDBJ Sequence Read Archive (DRA) under the accession numbers LC774870–LC774918 (Figure 4).

Water quality parameters for each sampling site are summarised in Table 1. All samples (K1–K8) contained relatively high concentrations of Na^+^, Cl^−^ and SO_4_^2−^, which are derived from sea salt carried by strong winds along the Antarctic coast (Tranter et al. 2005). Ponds in the off‐ice area (K1–K2) showed elevated levels of Ca^2+^ and Mg^2+^, possibly originating from minerals in metamorphic rocks (Hiroi et al. 1986). In contrast, supraglacial waters on the glacier (K4–K8) were characterised by generally low but still detectable concentrations of NO_3_^−^ and NH_4_^+^, whereas the other solutes (K^+^, Ca^2+^, Mg^2+^) were less abundant. The pH of the supraglacial waters (K4–K8) ranged from 6.00 to 6.50, which was slightly lower than that of ponds adjacent to the glacier (6.41–6.72; K1–K3).

**TABLE 1:** Concentrations of major dissolved ions (ppm) and pH of the sampling sites.

	pH	Na	K	Ca	Mg	NH_4_	Cl	NO_3_	SO_4_
K1	6.72	9.99	0.75	4.63	3.92	u.d.	20.08	0.03	17.98
K2	6.41	10.61	0.57	2.57	2.81	u.d.	26.25	0.06	6.51
K3	6.49	0.35	0.03	0.02	0.05	0.01	0.60	0.13	0.23
K4	6.02	0.16	0.02	u.d.	0.02	0.01	0.31	0.08	0.15
K5	6.50	0.17	0.02	u.d.	0.02	0.01	0.42	0.04	0.10
K6	6.18	0.17	0.02	u.d.	0.01	0.01	0.35	0.08	0.12
K7	6.14	0.21	0.03	u.d.	0.03	0.01	0.38	0.10	0.17
K8	6.00	0.27	0.01	u.d.	0.04	0.01	0.50	0.14	0.22

Spearman's rank correlation coefficients between the relative abundance of higher taxonomic groups and water quality parameters are shown in Table 2. Only statistically significant correlations (*p* < 0.05) are represented. These results highlight specific associations between the microbial community structure and the chemical characteristics of the sampling sites.

**TABLE 2:** Spearman's rank correlation coefficient between the eukaryotic community and water properties.

	pH	Na	K	Ca	Mg	NH_4_	Cl	NO_3_	SO_4_
Aquavolon		0.88	0.83	0.97	0.85		0.87		0.85
Cercozoa	0.81								
Chlorophyta		0.85	0.74		0.77		0.76		0.71
Choanoflagellatea				−0.78					
Chrysophyceae									
Ciliophora		0.88	0.93	0.90	0.85		0.81		0.85
Cryptophyta		0.80	0.82	0.97	0.85		0.79		0.85
Dictyochophyceae		0.80	0.82	0.97	0.85		0.79		0.85
Dinoflagellata		0.80	0.82	0.97	0.85		0.79		0.85
Fungi									
Streptophyta	−0.83		−0.76	−0.85			−0.71		
Others		0.85		0.76	0.72		0.86		0.71

*Note:* Only correlation coefficients with *p* values greater than 0.05 are shown. Positive correlations are shown in red, and negative correlations are shown in blue.

## 5 Discussion

### 5.1 Eukaryote Community Structure and Hydrologic Features

Structural differences among eukaryotic communities across the sampling sites appear to be partly related to the water quality parameters and the physical characteristics of site formation. Aquavolon, Ciliophora and Dictyochophyceae were detected exclusively in the more chemically enriched off‐ice ponds and were rarely observed in the supraglacial waters (Figure 2), suggesting that their distribution may be associated with higher concentrations of dissolved ions, particularly calcium (Table 2). In contrast, nitrate and ammonium concentrations were not strongly associated with community structure (Table 2). Despite the oligotrophic nature of glacial meltwaters, nitrogen was not completely depleted (Table 1), implying that microbial nitrogen fixation on glacier surface may supply sufficient bioavailable nitrogen to sustain primary producers and other microbial community members (Telling et al. 2011). Environmental differences within the glacier sites were also reflected in the distribution of dominant taxa. Small transient supraglacial puddles (e.g., K4 and K8) exhibited more acidic conditions (pH = 6.0) and lower overall ion concentrations, conditions that may support the growth of acid‐tolerant taxa such as Streptophyta (Hoham and Remias 2020). In contrast, Chrysophyceae were dominant in larger, more stable supraglacial lakes (K5 and K6), likely due to their adaptation to cold, slowly melting and relatively persistent aquatic environments (Remias et al. 2013). The diversification of eukaryotic communities at sites K5 and K6, despite having similar water chemistry to K4 and K8, suggests that certain ice/snow algae are capable of overwintering in water bodies that remain beneath the ice cover (Hotaling et al. 2021).

In the following sections, we discuss the genetic diversity of major taxonomic groups detected in the supraglacial waters (K4–K8); Chlorophyta, Chrysophyceae, Cercozoa, Streptophyta and Choanoflagellatea, in comparison with those found in the off‐ice area (K1–K3) as well as the other ice and snow fields previously published worldwide (Figure 4).

### 5.2 Chlorophyta

Chlorophyta was detected in all samples on Langhovde Glacier (K5–K8) as well as in the off‐ice area (K1–K3). Among this phylum, *Chloromonas* sp., which is one of the most widely detected snow algae with green or red colour (Hoham and Remias 2020), was found in the glacier samples (K5–K8) (ASV_008 and ASV_050). Both ASVs are close relatives of the cultured strain *Chloromonas muramotoi* (Matsuzaki et al. 2019) and *Chloromonas hindakii* (Procházková et al. 2019). ASV_008 matches the environmental samples (clone Otu003), collected from red snow in Alaska (Segawa et al. 2018), whereas ASV_050 matches the environmental samples (clones Otu005, Otu008 and Otu013) derived from red snow phenomena seen in the Antarctic Peninsula and Alaska (Segawa et al. 2018). ASV_036, detected in both K2 and K7, is also related to *Chloromonas* sp. and matches the environmental samples (clone Otu003) derived from glaciers in the Antarctic Peninsula and Alaska (Segawa et al. 2018).

ASV_032, found in the perennial supraglacial lake (K5) and off‐ice area (K1–K3), is identified as *Raphidonema* sp., which is a green snow alga widely distributed across both polar regions and mid‐latitude glaciers (Lutz et al. 2015; Segawa et al. 2023). ASV_032 shares 100% sequence identity with *Raphidonema sempervirens* CCCryo 011a‐99 isolated from green snow on the coast of Svalbard (Leya et al. 2003). It is closely related to environmental samples from glacial ice on the Tibetan Plateau (clone P60E‐26) (Zhang et al. 2009), red snow on Gulkana Glacier, Alaska (clone Otu006) (Segawa et al. 2018), volcanic soil in the Andes Mountains (clone B05_SE1B) (Costello et al. 2009) and glacial debris from Toklat Glacier, Alaska (clone TE101H) (Schmidt and Darcy 2015). Related sequences are also detected in the Langhovde off‐ice area (clones ANT_1–4) (unpublished). These results suggest that this group not only inhabits ice/snow but also cold, arid soil.

*Chlamydomonas* spp., another group of green or red snow algae commonly observed on mountain glaciers (Hoham and Remias 2020) was infrequently detected on Langhovde Glacier (K4–K8). Instead, *Chlamydomonas*‐related ASVs (ASV_005, ASV_059 and ASV_73) were found primarily in off‐ice areas (K1–K3). ASV_005 matches *Chlamydomonas* sp. ICE‐MDV, a strain isolated from the ice‐covered meromictic Lake Bonney in the Taylor Valley, Antarctica (Li et al. 2016; Raymond and Morgan‐Kiss 2017) as well as partial Chlorophyta genes (clones 1‐A6, 4‐G6 and 7‐B8) from sea ice in the Baltic Sea (Majaneva et al. 2012). ASV_059 matches *Chlamydomonas raudensis* strain CCAP 11/131 from Lake Bonney, Antarctica (Pocock et al. 2004) and *Chlamydomonas* sp. strain RCC2488 from the Arctic Beaufort Sea (Balzano et al. 2012). Previous studies have pointed to the cosmopolitan nature of the *Chlamydomonas* genotype, which includes environmental samples from lakes in coastal Antarctica (De Wever et al. 2009). Overall, *Chlamydomonas* spp. in Antarctica appear to be more adapted to water bodies in contact with rock surfaces rather than ice.

### 5.3 Chrysophyceae

Chrysophyceae sequence ASV_002 was detected in the perennial supraglacial lake (K5 and K6). This sequence matches *Hydrurus foetidus* isolated from torrent rivers draining Lake Finsevatn in Southern Norway (Klaveness and Lindstrøm 2011). *H. foetidus* is known to cause golden‐brown coloration of snow surfaces (Hoham and Remias 2020). ASV_002 also matches an uncultured environmental clone W8eE4 that was dominant in surface waters of Canadian High Arctic lakes (Charvet et al. 2012). Related sequences have been reported from oligotrophic waters in the off‐ice area of the Antarctic Peninsula (clone Boeck2, 27, 31) (Izaguirre et al. 2021), the margin of a semi‐permanent snowfield in Maritime Antarctica (clone Sva 10_3) and High Arctic Svalbard (clone Ant_26a) (Remias et al. 2013). Although Chrysophyceae in the polar region are highly diverse (Izaguirre et al. 2021), as is also indicated by the 18S rRNA gene analysis in this study, only ASV_002 was found on Langhovde Glacier and it was absent from the off‐ice area. Previous observations from Arctic and Antarctic fields indicate that *H. foetidus* prefers long‐lasting, slow‐melting snowpacks (Remias et al. 2013). It is presumed that this alga may have been transported atmospherically from distant locations, colonising the relatively persistent supraglacial lakes (K5 and K6) on the glacier surface.

### 5.4 Cercozoa

Three ASVs classified as Cercozoa (ASV_017, 022 and 064) were detected exclusively on the glacier, whereas five ASVs (ASV_016, 051, 077, 081 and 084) were found in both glacier and off‐ice sites. They are genetically similar, except for ASV_081, which is closely related to *Heteromita* spp. (Brabender et al. 2012; Ekelund et al. 2004). *Heteromita* spp. have been reported from glaciers in other regions (García‐Descalzo et al. 2013; Yakimovich et al. 2020) and appear to be associated with snow and ice environments. ASV_081 matches environmental clones TE203A and TE201F, both detected in soil samples around Toklat Glacier, Alaska (Schmidt and Darcy 2015). These results suggest the potential similarities between Cercozoa communities on Langhovde Glacier and Toklat Glacier. However, the other ASVs identified in this study also show close similarity to multiple strains of *Bodomorpha* spp. and *Sandonidae* spp., which have been reported from non‐cryospheric environments. This indicates that the amplified region may be insufficient to resolve the phylogeny of Cercozoa at the genus level, and analysis using alternative genetic markers is needed to fully support these interpretations.

### 5.5 Streptophyta

Streptophyta was the dominant taxon (> 90%) in K4 and K8, which are located near the eastern and western margins of Langhovde Glacier and exist only during summer. It was also predominant (23%–46%) in the other supraglacial waters (K5, K6 and K7), but was rarely detected in the off‐ice sites (K1–K3). The Streptophyta community on the glacier consisted primarily of ASV_001 and partly of ASV_082. ASV_001 matches *Ancylonema nordenskioeldii* WP211, isolated from the surface of Gurgler Glacier (Procházková et al. 2021; Remias et al. 2012), as well as *Ancylonema nordenskioeldii* CCCryo BS_0001–2000 from North‐western Spitsbergen (Leya et al. 2003) and *Ancylonema alaskanum* CCCryoDEU: BS_0002–2009 from glacier ice in Austria (Remias et al. 2012). Related environmental sequences of ASV_001 are also reported from glaciers in the Himalayas (clones TE101F, TE105C and TE105D) (Schmidt and Darcy 2015) and from snow in the Canadian High Arctic (clone DGe1) (Harding et al. 2011). *Ancylonema* spp. are distributed worldwide and are known to cause green‐ or grey‐coloured surfaces on ice and snow (Hoham and Remias 2020). Although ASV_082 did not match any sequences in the GenBank database with 100% identity, it showed similarity to *Mougeotia* sp. CCAC 0197 (Cheng et al. 2019), *Mesotaenium* cf. *chlamydosporum* strain M‐2155 (Gontcharov et al. 2004) and *Fottea pyrenoidosa* strain SAG 1.88 (Gontcharov and Melkonian 2010). These genera are sometimes found among widespread ice/snow algae in both hemispheres (Lutz et al. 2018; Takeuchi 2013). Except for a single sequence (ASV_070) with a tiny number of reads, no Streptophyta was detected in the off‐ice area. This suggests that ASV_001 and ASV_082 found on Langhovde Glacier may have originated from distant regions via atmospheric transport.

### 5.6 Choanoflagellatea

Choanoflagellatea sequences detected on the glacier (ASV_7, 11 and 12) were closely affiliated with the family Salpingoecidae. However, they differed from previously reported sequences at the genus level, suggesting that they may represent previously undescribed species. Salpingoecidae is widely distributed in marine and freshwater environments, yet reports from glacial habitats are scarce. One exception is *Monosiga* spp., which has been detected in glacial meltwater puddles in Northwest Greenland (IkÄvalko et al. 1996). Choanoflagellates are heterotrophic protists and important bacterial grazers in many aquatic ecosystems (Boenigk and Arndt 2002); therefore, they may play a significant ecological role in the microbial food web of supraglacial waters on Langhovde Glacier. However, phylogenetic classification of Choanoflagellatea based on the 18S rRNA gene remains limited (Nitsche et al. 2011), and additional molecular and ecological studies are required to further elucidate their diversity, function and distribution in glacial environments.

## 6 Conclusion

This study analysed environmental DNA from supraglacial waters on Langhovde Glacier, East Antarctica, using 18S rRNA gene sequencing with universal primers targeting eukaryotes. The results were compared with those of the same analyses conducted in ponds in the off‐ice area to assess local community composition and biodiversity patterns. A wide variety of eukaryotic taxa, including Chlorophyta, Chrysophyceae, Cercozoa, Streptophyta and Choanoflagellatea, were detected on Langhovde Glacier. Among them, microbial diversity was significantly higher in large perennial lakes compared with small transient puddles, which were predominantly composed of Streptophyta. Some sequences matched previously reported coloured ice/snow algae communities worldwide but were rarely observed in the aquatic ecosystems of the nearby off‐ice area. Although this study focused exclusively on the 18S rRNA gene region and further investigation is needed, the findings suggest that microbial community composition on Langhovde Glacier may vary interannually and evolve independently from the adjacent off‐ice ecosystems, reflecting localised aquatic environmental controls.

## Author Contributions

**Hiroto Kajita:** conceptualization, writing original draft, formal analysis, investigation. **Ken Kondo:** writing, review and editing, resources, visualization. **Shin Sugiyama:** writing, review and editing, resources, project administration. **Yasuhide Nakamura:** writing, review and editing, data curation. **Sakae Kudoh:** data curation. **Koji Umeda:** supervision, funding acquisition.

## Conflicts of Interest

The authors declare no conflicts of interest.

## Supporting information


**Data S1.** emi470117‐sup‐0001‐supinfo.

## Data Availability

Data are archived in the DDBJ Sequence Read Archive (DRA) database and Supporting Information file.
